# Histone H3 Serine 57 and Lysine 56 Interplay in Transcription Elongation and Recovery from S-Phase Stress

**DOI:** 10.1371/journal.pone.0010851

**Published:** 2010-05-26

**Authors:** Aamir Aslam, Colin Logie

**Affiliations:** Department of Molecular Biology, Nijmegen Centre for Molecular Life Sciences, Radboud University Nijmegen, The Netherlands; Oregon State University, United States of America

## Abstract

**Background:**

Acetylation of lysine 56 of histone H3 plays an important role in the DNA damage response and it has been postulated to play an as yet undefined role in transcription, both in yeast and in higher eukaryotes. Because phosphorylated human histone H3 serine 57 peptides have been detected by mass spectrometry we examined whether H3-S57 phosphorylation interplays with H3-K56 acetylation *in vivo*.

**Methodology/Principal Findings:**

To explore the physiological role of H3-S57, H3-K56 was mutated to mimic constitutively (un)acetylated forms of H3-K56 and these were combined with constitutively (un)phosphorylated mimics of H3-S57, in yeast. A phosphorylated serine mimic at position 57 lessened sensitivities to a DNA replication fork inhibitor and to a transcription elongation inhibitor that were caused by an acetylated lysine mimic at position 56, while the same substitution exacerbated sensitivities due to mimicking a constitutive non-acetylated lysine at position 56. Strikingly, opposite results were obtained in the context of a serine to alanine substitution at position 57 of histone H3.

**Conclusions/Significance:**

The phenotypes elicited and the context-dependent interplay of the H3-K56 and -S57 point mutations that mimic their respective modification states suggest that serine 57 phosphorylation promotes a nucleosomal transaction when lysine 56 is acetylated. We speculate that histone H3-S57 couples H3-K56 acetylation to histone quaternary structures involving arginine 40 on histone H4 helix 1.

## Introduction

Lysine 56 (H3-K56) occupies a strategic location at the ends of the two histone H3's α-N helices within the nucleosome particle and likely plays a pivotal role in nucleosomal DNA dynamics by making a water mediated hydrogen bond with the DNA that enters and exits the nucleosome [1], [2]. A H3-K56 acetylation cycle has been shown to play a crucial role in the capacity of yeast to resist the toxic effects of compounds that cause double strand DNA breaks [3]–[5]. Furthermore, H3-K56 acetylation has also been implicated in gene expression in yeast and in higher eukaryotes [6]–[8]. A previously unknown yeast histone acetyl transferase (HAT), Rtt109p, bearing structural resemblance to the mammalian p300/CBP HATs [9], [10] has been demonstrated to be responsible for H3-K56 acetylation [11], [12]. In mammalian cells, both CBP and GCN5 have been put forward as H3-K56 HATs [13], [14]. Interestingly, H3-K56 appears to be targeted by HATs prior to being deposited onto DNA, by virtue of newly synthesised H3 being in complex with the histone chaperone Asf1p [15]–[17] or its mammalian homolog ASF1A [13]. The association of H3-K56 acetylation with the nucleosome deposition machinery mechanistically explains how this modification associates with progressing replication forks and with promoter nucleosomes that display high turnover [6], [18]. In mammalian cells, SIRT1 and SIRT6 have been proposed as H3-K56 deacetylases [13], [19], [20], [21]. In yeast, removal of the H3-K56ac mark is performed by the sirtuin-related enzymes Hst3p and Hst4p [22], [23]. Hst4p is responsible for keeping H3-K56ac levels low in G1 whilst Hst3p deacetylates H3-K56ac in the wake of replication forks [22]. When S-phase DNA damage is sensed, yeast *HST3* mRNA levels are reduced in a Mec1p DNA damage checkpoint kinase dependent fashion during G2, thus prolonging the presence of chromosomal H3-K56ac [23]. Exactly how H3-K56 facilitates recovery from double strand DNA breaks is still unknown [24], [25] although it is known that K56 acetylation increases the affinity of histone H3 for the histone chaperones Rtt106p and CAF-1 [26] as well as increasing the ‘breathing’ of DNA entering and exiting the nucleosome 7-fold [27].

It was discovered through mass spectrometry analysis that H3-S57, the serine adjacent to H3-K56, can be phosphorylated in mammalian cells (H3-S57ph, M. Vermeulen personal communication). However, to date the presence of multiple mammalian cell histone H3 genes has prohibited functional mammalian histone mutagenesis studies. We therefore performed a functional mutagenesis study involving H3-K56 and H3-S57 in yeast, even though to date we have not detected H3-57ph in this organism. We assessed proliferation in the presence of the clastogen methylmethane sulfonate (MMS), the nucleotide reductase inhibitor hydroxyurea (HU) and the transcription inhibitor 6-azauracil (6-AU). We found that ablating serine 57 or mimicking its phosphorylation affects cells differentially upon combination with constitutively acetylated and non-acetylated lysine 56 mimics, demonstrating functional interplay between the residues at positions 56 and 57 of histone H3. Specifically, we find that sensitivity to MMS, HU and 6-AU is partially alleviated when lysine 56 and serine 57 modified forms are mimicked together. Conversely, constitutively mimicking only one modified residue exacerbated sensitivity to the inhibitors. Furthermore, and contrary to replication inhibition, transcription inhibition by 6-AU does not appear to be modulated substantially by serine 57 substitutions when lysine 56 function is ablated by substitution to alanine, indicating a differential requirement of replication and transcription on H3 serine 57 function.

## Methods

### Attempted detection of yeast H3-S57ph

Human H3-S57ph was detected in *C. elegans* (www.phosida.com) [30] and in HeLa cell extracts that were highly enriched for histones with the methodology employed to detect H3-T45ph (M Vermeulen personal communication, [28]). H3-S57ph was not detected in the synchronized cell cycle phosphoproteome data reported by Olsen *et al.* [29], indicating that it is difficult to detect and therefore probably not abundant in human cells. In yeast we have not applied the titatnium dioxide bead technique to enrich phosphorylated tryptic peptides, and using older methods we never detected H3-S57ph [3]. In an attempt to obtain indirect evidence for H3-S57ph in yeast we immunized 2 rabbits with H3-S57ph bearing peptides. Unfortunately, peptide dot blots did not reveal phopho-epitope specific antibodies. To date and to the best of our knowledge there is therefore as yet no evidence demonstrating the existence of H3-S57ph in *S. saccharomyces* [31].

### Yeast Strains, Plasmids, and Media

A list of yeast strains is provided (Table 1). Site-directed mutagenesis of plasmid p*HHT2*-*HIS3* was performed as described [3] and was confirmed by sequencing the entire gene. Compounds were added to YEPD (1% yeast extract, 2% bacto-peptone, 2% dextrose) agar or liquid medium to the final concentrations indicated in the figure legends; hydroxyurea (HU; Sigma), methyl methanesulfonate (MMS; Acros Organics), formamide (Fluka Biochemicals), 6-azauracil (6-AU; Sigma). The operational 5-fluoroorotic acid (5-FOA; ICN Biochemicals) concentration was 0.1% (w/v). The α-factor pheromone peptide (synthesized in-house) was used to make 10 µg/ml solutions. A Stratagene ultra violet (UV) Stratalinker was used in ‘energy mode’ to achieve 100 J/m^2^ of 254 nm irradiation. Yeast cells were grown into log phase in YEPD (OD_600_ of 0.2) and used for cell cycle synchronization or spotted as 5-fold serially diluted 5 µl droplets on the indicated YEPD plates and photographed after 3 days at 30°C.

**Table 1:** *Saccharomyces cerevisiae* strains used in this study.

Name	Genotype	Reference short hand
YN 1037	*MATa*; *his3*Δ*1*; *leu2*Δ*0*; *LYS2*; *met15*Δ*0*; *ura3*Δ*0*; *Euroscarf* BY4741	Wild type, *ura3*
YN 1375	*MATa*; *his3*Δ*1*; *leu2*Δ*0*; *LYS2*; *met15*Δ*0*; *ura3*Δ*0*; *YBR010w::kanMX4*, *YNL031c::kanMX4*, *[pMR366-HHT2-URA3]* −− *hht1*Δ ; *hht2*Δ−−	[3] (WT-URA3)
YN 1392	*MATa*; *his3*Δ*1*; *leu2*Δ*0*; *LYS2*; *met15*Δ*0*; *ura3*Δ*0*; *YBR010w::kanMX4*, *YNL031c::kanMX4*; *[pHHT2_K56A-HIS3]* −− *hht1*Δ ; *hht2*Δ−−	[3]
		AS
YN 1393	*MATa*; *his3*Δ*1*; *leu2*Δ*0*; *LYS2*; *met15Δ0*; *ura3*Δ*0*; *YBR010w::kanMX4*, *YNL031c::kanMX4*; *[pHHT2_K56R-HIS3]* −− *hht1*Δ ; *hht2*Δ−−	[3]
		RS
YN 2168	*MATa*; *his3*Δ*1*; *leu2*Δ*0*; *LYS2*; *met15Δ0*; *ura3*Δ*0*; *YBR010w::kanMX4*, *YNL031c::kanMX4*; *[pHHT2_S57A-HIS3]* −− *hht1*Δ ; *hht2*Δ−−	This work
		KA
YN 2170	*MATa*; *his3*Δ*1*; *leu2*Δ*0*; *LYS2*; *met15Δ0*; *ura3*Δ*0*; *YBR010w::kanMX4*, *YNL031c::kanMX4*; *[pHHT2_S57E-HIS3]* −− *hht1*Δ ; *hht2*Δ−−	This work
		KE
YN 2172	*MATa*; *his3*Δ*1*; *leu2*Δ*0*; *LYS2*; *met15Δ0*; *ura3*Δ*0*; *YBR010w::kanMX4*, *YNL031c::kanMX4*; *[pHHT2_K56R, S57A-HIS3]* −− *hht1*Δ ; *hht2*Δ−−	This work
		RA
YN 2174	*MATa*; *his3*Δ*1*; *leu2*Δ*0*; *LYS2*; *met15Δ0*; *ura3*Δ*0*; *YBR010w::kanMX4*, *YNL031c::kanMX4*; *[pHHT2_K56R, S57E-HIS3]* −− *hht1*Δ ; *hht2*Δ−−	This work
		RE
YN 2176	*MATa*; *his3*Δ*1*; *leu2*Δ*0*; *LYS2*; *met15Δ0*; *ura3*Δ*0*; *YBR010w::kanMX4*, *YNL031c::kanMX4*; *[pHHT2_K56Q-HIS3]* −− *hht1*Δ ; *hht2*Δ−−	This work
		QS
YN 2178	*MATa*; *his3*Δ*1*; *leu2*Δ*0*; *LYS2*; *met15Δ0*; *ura3*Δ*0*; *YBR010w::kanMX4*, *YNL031c::kanMX4*; *[pHHT2_K56Q, S57A-HIS3]* −− *hht1*Δ ; *hht2*Δ−−	This work
		QA
YN 2180	*MATa*; *his3*Δ*1*; *leu2*Δ*0*; *LYS2*; *met15Δ0*; *ura3*Δ*0*; *YBR010w::kanMX4*, *YNL031c::kanMX4*; *[pHHT2_K56Q, S57E-HIS3]* −− *hht1*Δ ; *hht2*Δ−−	This work
		QE
YN 2182	*MATa*; *his3*Δ*1*; *leu2*Δ*0*; *LYS2*; *met15Δ0*; *ura3*Δ*0*; *YBR010w::kanMX4*, *YNL031c::kanMX4*; *[pHHT2_K56A, S57A-HIS3]* −− *hht1*Δ ; *hht2*Δ−−	This work
		AA
YN 2184	*MATa*; *his3*Δ*1*; *leu2*Δ*0*; *LYS2*; *met15Δ0*; *ura3*Δ*0*; *YBR010w::kanMX4*, *YNL031c::kanMX4*; *[pHHT2_K56A, S57E-HIS3]* −− *hht1*Δ ; *hht2*Δ−−	This work
		AE
YN 2186	*MATa*; *his3*Δ*1*; *leu2*Δ*0*; *LYS2*; *met15Δ0*; *ura3*Δ*0*; *YBR010w::kanMX4*, *YNL031c::kanMX4*; *[pHHT2-HIS3]* −− *hht1*Δ ; *hht2*Δ−−	This work
		KS (WT-HIS)

### Flow Cytometry Analysis

Cellular DNA content was determined as described [32] using 1 µM sytox green (Molecular Probes) and a BD Biosciences calibur fluorescence activated cell sorter.

## Results

### Lack of dominant effects of histone H3 lysine 56 and serine 57 mutations

**Figure 1:**
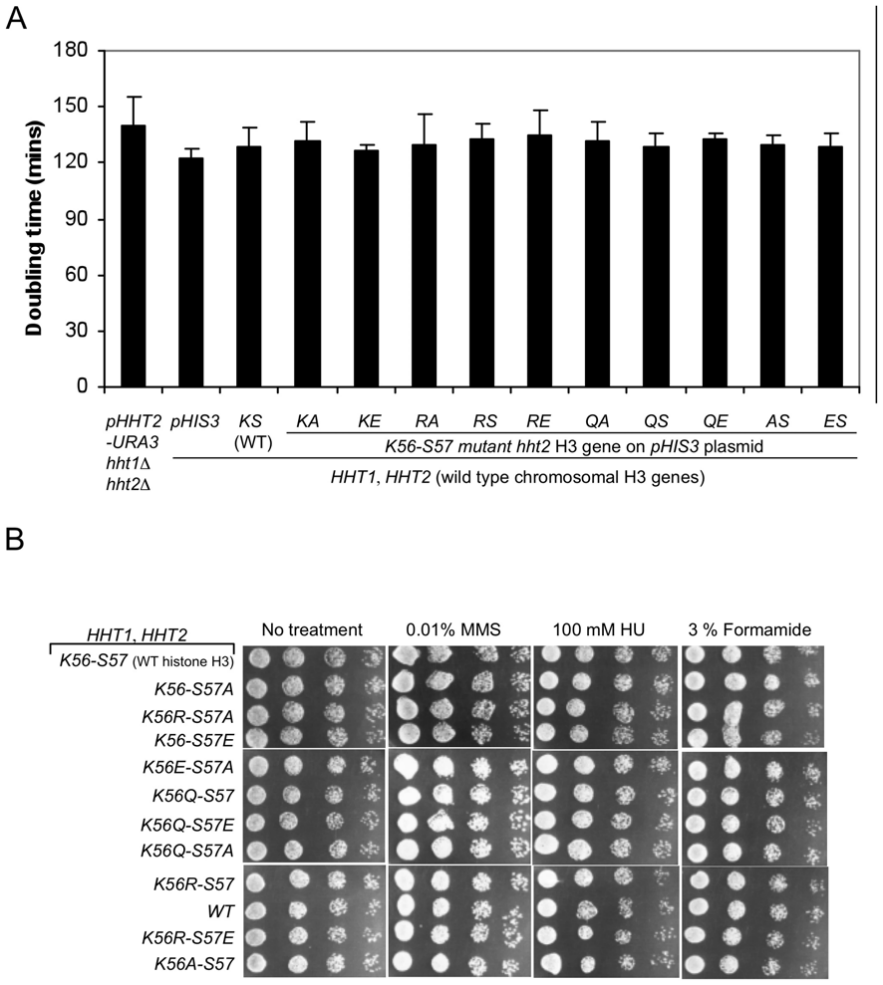
Lack of dominant negative effects of the H3-K56/H3S57 mutant histone genes. The plasmid-borne H3-K56 (Q, R) and -S57 (A, E) *hht2* point mutations were analyzed for dominant negative effects in YN1038, a strain harboring wild type chromosomal copies of the *HHT2* and *HHT1* yeast histone H3 genes. Neither growth rates measured at 30°C (A) and nor clone sizes determined on plates containing methyl methanesulfonate, hydroxyurea or formamide (B) revealed any dominant effects of the mutant histone H3 genes when wild type yeast histone H3 was present.

**Figure 2:**
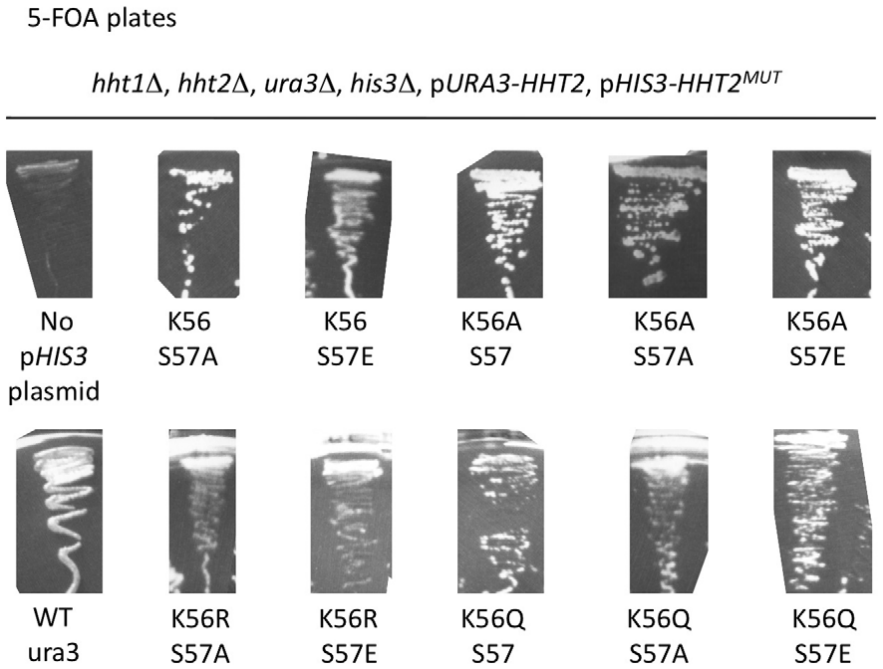
Recovery rates of yeast clones harboring H3-K56/S57 point mutations. YN1375-derived yeast strains harboring the wild type allele of the yeast histone H3 gene *HHT2* on a *URA3* gene-bearing plasmid as well as the indicated *hht2* allele on a *HIS3*-gene bearing plasmid were streaked-out on 5-FOA plates to select for cells that had lost the *URA3*-bearing plasmid [3]. Note that 5-FOA resistant clones emerged at similar frequencies for all the strains except for the positive control strain (*WT*, *ura3*) that lacked a *URA3* gene to begin with and the negative control strain that lacked a histone H3 gene borne on a *HIS3* plasmid (*hht1*Δ, *hht2*Δ, *pURA-HHT2*). The *hht2-K56R* allele is not shown here but an identical experiment was described previously [3].

In order to explore whether H3-S57 interplays with H3-K56 acetylation we employed a *Saccharomyces cerevisiae* yeast strain where both endogenous H3 genes were deleted. The yeast were kept alive with a counter-selectable plasmid driving expression of wild type histone H3 [3]. Mutations were introduced on a second H3 expression plasmid and phenotypes were assessed in the presence or absence of the plasmid bearing the wild type H3 allele. Mutations replaced lysine 56 with alanine, arginine or glutamine, eliminating lysine function, mimicking an non-acetylated lysine or mimicking a constitutively acetylated lysine, respectively. These were then combined with serine 57 substitutions into alanine, or glutamate, eliminating serine function or mimicking a phosphorylated serine, respectively. We did not detect any dominant effects at the level of growth rates or any of the tested phenotypes in yeast harboring both the wild type and any mutant version of histone H3 (Figure 1). Furthermore, and in contrast to introduction of a glutamate at position 56 which is lethal [3], [33], all of the above single and double mutations – including a glutamate at position 57 – could support life, as we obtained viable mutant yeast clones with similar frequencies (Figure 2).

### Serine 57 substitutions impair the DNA damage response

**Figure 3:**
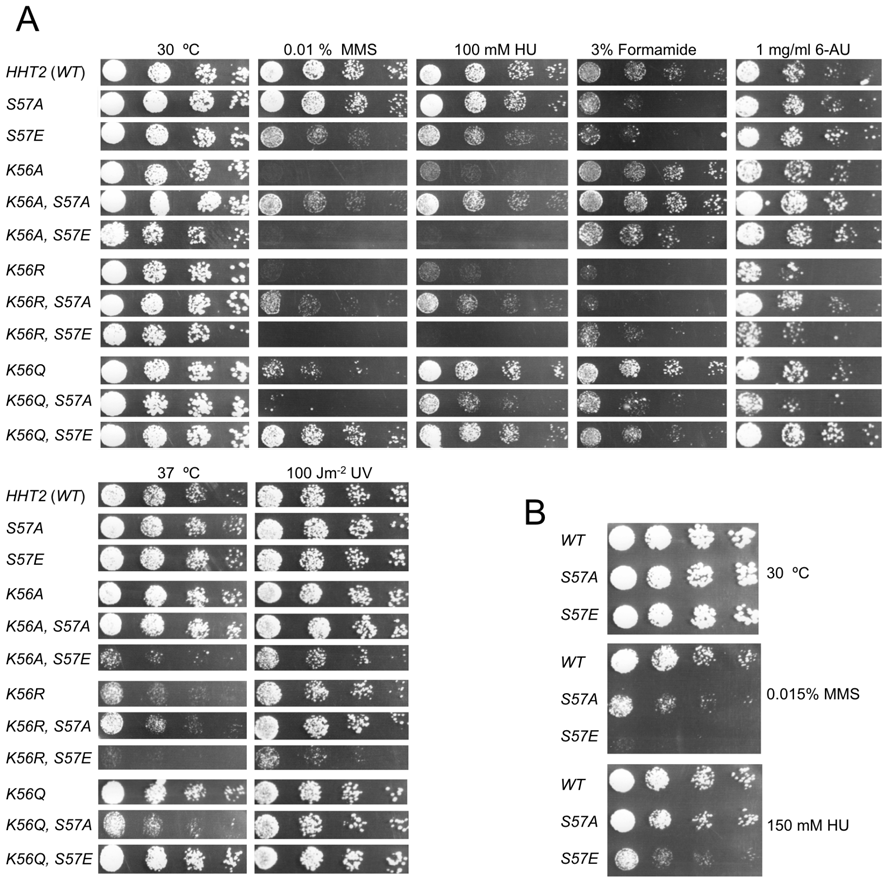
Interplay between H3-K56 and H3-S57 mutations. A. Five-fold serial dilutions of the indicated mutant strains were analyzed on the indicated YEPD plates. One representative experiment out of at least three is shown. Note the formamide sensitivity of both single H3-S57 mutants and also the inverse MMS, HU and 6-AU hypersensitivity relations between the H3-S57A and H3-S57E mutations in the contexts of the H3-K56R and H3-K56Q point mutations. B. Wild type H3, H3-S57A and H3-57E were tested as in (A) but using higher concentrations of MMS and of HU.

To uncover functional relations, we assessed phenotypes induced by compounds that target DNA damage to S-phase (MMS, HU), generate pyrimidine dimers (UV), disrupt hydrogen bonding (formamide) or that inhibit transcription (6-AU). Mutation of H3-S57 to alanine or glutamate had no effect on sensitivity to 6-AU, UV or growth at higher temperature (Figure 3A) and sensitized yeast to formamide to a similar extent. Interestingly however, the H3-S57E mutant was more sensitive than the H3-S57A mutant to DNA damage caused by MMS (Figure 3A). Additional experiments with higher doses of MMS and HU showed that both of the single H3-S57 substitutions were sensitive to S-phase stress. However, while H3-S57E was sensitive to both HU and MMS, S57A was not sensitive to HU, even at a higher concentration (Figure 3B). These results suggest that histone H3 serine 57 has some role when yeast cells face S-phase stress induced by MMS [34].

### Stress-specific suppression and enhancement of lysine 56 mutations by serine 57 mutations

If particular mutations at histone H3-K56 and H3-S57 simply affect growth rate, we would expect the profile of drug sensitivities to reflect this. However, if the modification status of these residues were to interplay under given stress conditions then we would predict that single and double mutants will behave differently than predicted from the product of the effects of the single mutations. What we observed is indeed the latter scenario (Figure 3A).

Relative to single lysine 56 substitutions by themselves, yeast were more sensitive to S-phase chromosome damage caused by MMS when constitutively non-acetylated lysine (K56R) was mimicked along with a H3-S57ph mimic (S57E, Figure 3A). In the converse situation, where a H3-K56ac mimic (K56Q) was combined with a loss of serine at position 57 (S57A) the yeast were more sensitive to MMS also. In contrast, the constitutive H3-S57ph mimic mitigated hypersensitivity to MMS due to a constitutive H3-K56ac mimic (Figure 3A). Furthermore, the deleterious effects of the H3-K56A mutation on DNA damage sensitivity were exacerbated by the H3-S57ph mimic (S57E) and strongly suppressed by the S57A substitution, similar to the interplay with the H3-K56R mutation (Figure 3A). A specific role for H3-S57 in recovery from DNA alkylation induced by MMS is underscored in the H3-K56Q background by the fact that the alleviating effect of mimicking H3-S57ph alongside H3-K56ac did not extend to exposure to HU or formamide (Figure 3A).

Some aspect of this interplay may extend to transcriptional elongation since the K56Q-S57E combination also alleviated K56Q induced growth inhibition on 6-AU containing growth medium. Notably however, when H3-K56 function was ablated by substitution to alanine (K56A) the serine 57 substitutions had no discernible effect on 6-AU sensitivity, suggesting that cells do not need to invoke H3-S57 function to promote transcription elongation when a hydrophobic residue is present at position 56. Altogether, these results demonstrate that the residues at positions 56 and 57 of histone H3 can interplay functionally, as the effects of the point mutations are not individually additive.

### The H3-S57E phosphorylation mimic prolonges the G2/M cell cycle arrest induced by MMS

**Figure 4:**
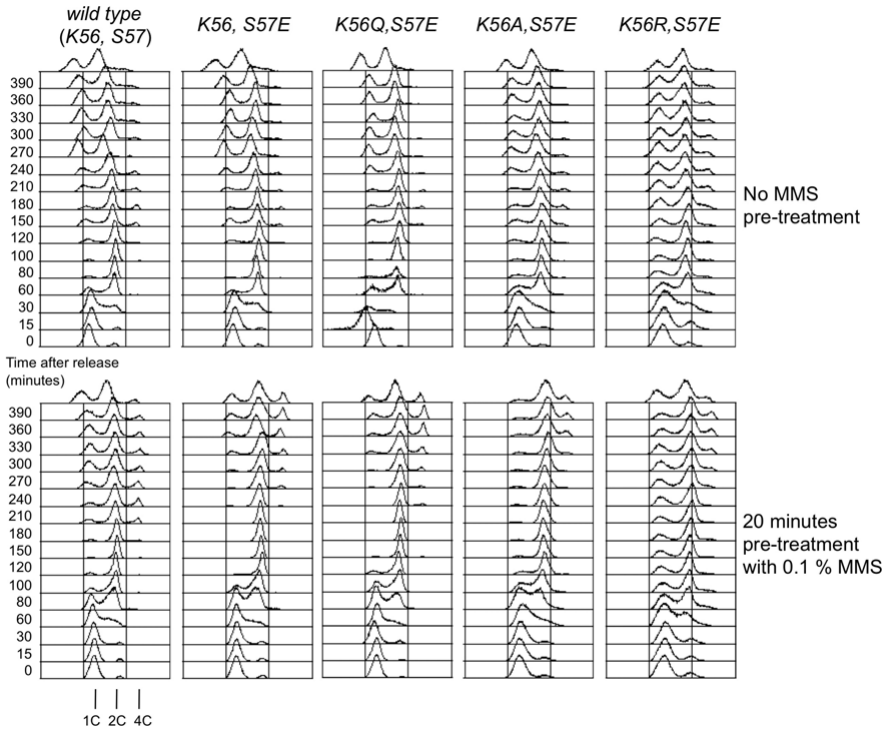
Prolonged G2/M delay upon brief exposure of H3-K56R/A/Q-S57E mutants to MMS in G1. The indicated yeast strains were arrested in G1 with mating pheromone and divided into two samples that were exposed (bottom) or not (top) to 0.1% MMS for 20 minutes prior to release from a G1 arrest into complete growth medium. Cell cycle progression was measured as a function of cellular DNA content in samples collected at the indicated time.

Hypotheses as to the nature of the cellular defects leading to the above observations include an inability of cells to mount a cell cycle arrest or to resume the cell cycle in a timely fashion after such an arrest [35]. To reveal MMS induced cell cycle phenotypes we employed H3-S57E mutants, including double mutants that displayed exacerbating (K56A-S57E, K56R-S57E) and mitigating (K56Q-S57E) interactions with H3-K56 mutations. The yeast were synchronized in the G1 phase of the cell cycle using alpha mating type pheromone, pulsed with a high level of MMS (0.1%) for 20 minutes and then released into the cell cycle and monitored for DNA content (Figure 4).

All the strains performed an efficient G1 arrest upon exposure to pheromone. Control cells expressing wild type or mutant plasmid-borne histone H3 genes entered S-phase 30 minutes after release from the pheromone arrest. Completion of DNA replication occurred 30 minutes later for the wild type and the H3-K56E mutant whilst it took some 20 minutes longer for the three double mutants (Figure 4 and data not shown). New G1 cells, indicative of completion of mitosis, could be detected some 40 minutes later for all the strains. These data are in keeping with bulk culture doubling times of about 130±5 minutes for wild type cells, 140±5 for the H3-K56Q-S57E mutant and 160±9 minutes for the H3-K56R-S57E mutant. As we repeatedly observed that the H3-K56A-S57E and H3-K56R-S57E cultures harbored a small subpopulation of cells that did not initiate S-phase synchronously (Figure 4 and data not shown) it is likely that the higher bulk doubling time, as well as the slower colony growth on agar plates observed for the H3-K56A-S57E and H3-K56R-S57E strains (Figures 2, 3) was partly due to subpopulations of non-cycling cells.

Exposure to MMS whilst cells were arrested in G1 resulted in a 30 minute delay in the initiation of S-phase in all the strains and a further S-phase completion delay of about 10–20 minutes, as revealed by the disappearance of cells with less than a double genome complement (Figure 4, compare top and bottom panels). The wild type cells exposed to MMS then delayed in G2/M for an additional 60 minutes before completing mitosis, as measured by the appearance of new G1 cells. On the other hand, all the mutants, including the single H3-S57E mutant, displayed an additional 4 hour G2/M delay, indicating that all the mutants were unable to complete mitosis efficiently. As such, it would therefore appear that all the H3-S57E mutants display the same cell cycle defect which results in a prolonged G2/M delay, irrespective of their survival rates which differed >5 fold (Figure 3, data not shown).

In this particular experiment, re-replication in the absence of mitotic completion took place in all the cultures, including wild type cells, as indicated by the appearance of a small sub-population of cells apparently harboring 4 genome complements (Figure 4). Cells having completed re-replication are apparent by 180 minutes for wild type, 240 minutes for H3-S57E and H3-K56Q-S57E cultures and 300 minutes for H3-K56A/R-S57E mutants (Figure 4). This last observation qualitatively parallels the relative survival capacity of the mutants upon MMS exposure, whereby those displaying re-replication last are those who survive least.

Our cell cycle analyses thus suggest that it is not a failure to arrest, nor to eventually attempt to resume the cell division cycle in the face of replication fork stress that underlies the very poor survival of the H3-K56A-S57E and H3-K56R-S57E mutants upon exposure to MMS (Figure 3) but rather a failure to perform a histone H3-dependent chromatin transaction required to successfully recover from exposure to clastogens such as MMS [34].

## Discussion

The results we present above indicate that histone H3 serine 57, which like lysine 56 is an evolutionarily fully conserved histone residue, plays a dynamic role in the response to replication fork-linked DNA damage repair [36]–[38]. Although not ruling out a direct role in transcription elongation or replicational DNA break repair, our cell cycle data is consistent with the conjecture that the H3-K56 acetylation cycle plays an important role in signalling the completion of repair through chromatin reassembly at sites of double strand breaks [35] and implicates H3-S57 in the same pathway.

**Figure 5:**
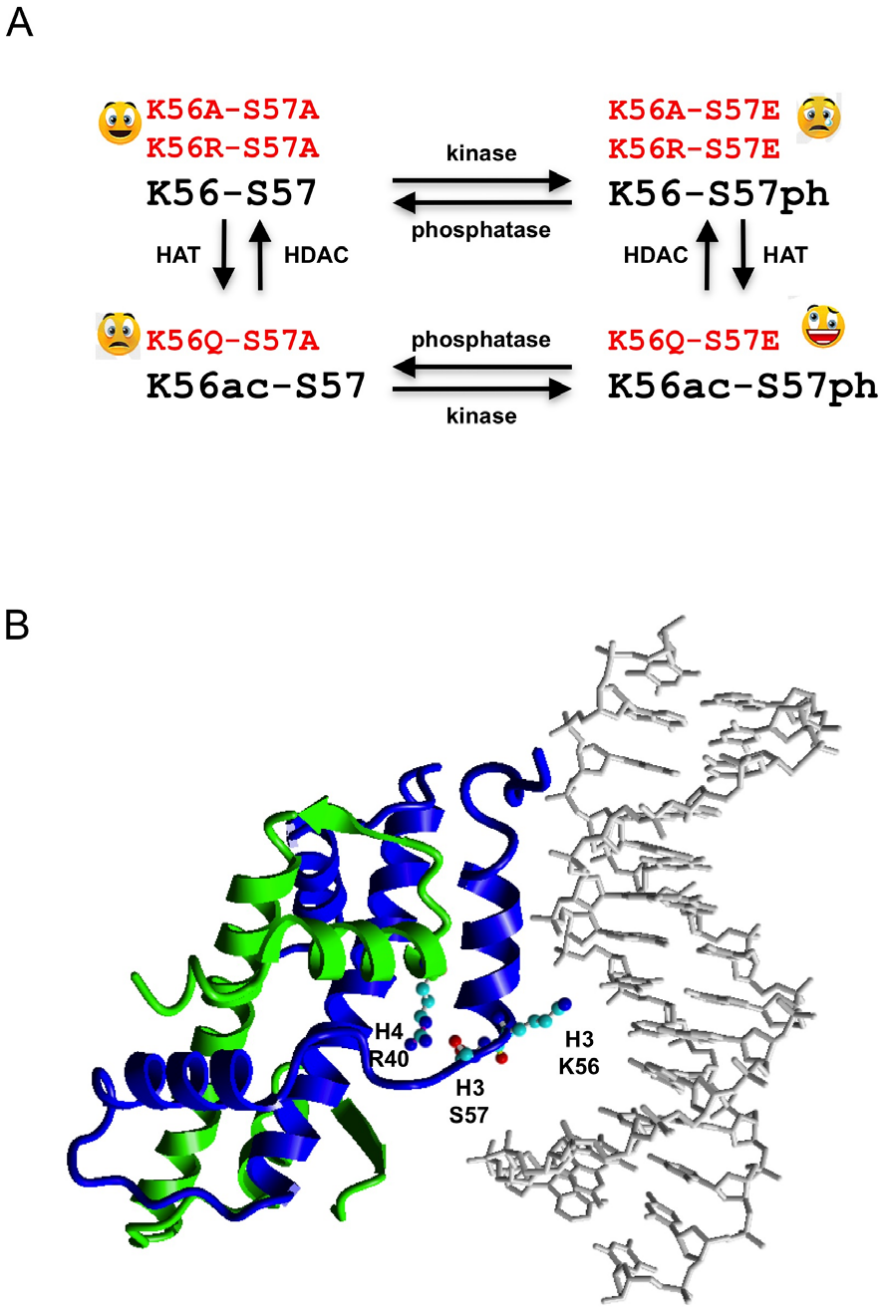
Regulatory and atomic context of histone H3 serine 57. A. Theoretical scheme imbricating the acetylation cycle of H3-K56 with the putative phosphorylation cycle of H3-S57. Double point mutations introduced to constitutively mimic the 4 possible modification states are indicated in red. The observed fitness upon induction of S-phase double strand breaks by MMS (Figure 3A) is shown as smileys next to the mutations. B. Ribbon view of one nucleosomal molecule of histone H3 (blue) and of histone H4 (green) as well as the first 14 base pairs of nucleosomal DNA, displaying the H3-K56, H3-S57 and H4-R40 residue atoms as ball and stick. H3-K56 makes a water mediated contact with bp 9 of the DNA and H3-S57 is linked by a hydrogen bridge to H4-R40 on histone H4 helix 1. This figure was built using Yasara (http://www.yasara.org) and PDB file 1ID3 [2]. The same hydrogen bridges are visible in all the nucleosome crystal structures we examined [1], [2], [54].

We have not ruled out the possibility that the serine 57 substitutions we employed affect the capacity of yeast to acetylate or subsequently deacetylate H3-K56. However, this does not affect our conclusions much as they are based on the phenotypes of double mutants that constitutively mimic the (un)modified state of both lysine 56 and serine 57, entirely bypassing the need to modify H3-K56 (Figure 5A). To mimic H3-S57ph we substituted glutamate for serine 57. Glutamate closely mimics the length of the R chain of a phosphorylated serine which is about 4.8 Å from Cα to the furthest oxygen. An alternative would have been to substitute aspartate [39]. We predict that substituting aspartate would result in less pronounced effects because it is shorter by about 1.1 Å. If our results were to be duplicated using aspartate mutants obtained from site directed [39] or random mutagenesis [33] this would strengthen the tentative conclusions we draw below. Positive identification of H3-S57 putative kinase and phosphatase activities [30] would shed more light on the role of H3-S57 in chromatin metabolism. Until then alternative interpretations of our results that do not invoke phosphorylation of H3-S57 must also be considered.

As could be expected for an important nucleosomal moiety and similarly to what we observed with single H3-K56 point mutations [3], the H3-S57 substitutions we studied also induced phenotypes under other stress conditions such as exposure to formamide (Figure 3), and even UV in the case of the H3-K56R-S57E and K56A-S57E double mutants (Figure 3).

Our analysis suggests that a dynamic interaction between lysine 56 and serine 57 is important for survival to clastogenic agents as we demonstrate that yeast are much more sensitive to DNA damage when only H3 lysine 56 or only serine 57 modified forms are constitutively mimicked (K56R-S57E/K56A-S57E; K56Q-S57A) than when both (K56Q-S57E) or neither (K56R-S57A/K56A-S57A) are (Figures 3 and 5A). This context dependency suggests that serine 57 phosphorylation would promote a nucleosomal transaction only when lysine 56 can be acetylated (Figure 5A).

The phenotypic interplay between amino acids at positions 56 and 57 of histone H3 also points to a specific function in transcription elongation, as has been proposed previously [7], [8], [17], [40], [41]. However, and contrary to DNA damage, a role for serine 57 in transcription elongation was only apparent in our experiments when the DNA strands entering and exiting the nucleosome are constrained by a glutamine or an arginine at position 56, since in the context of exposure to 6-AU, entirely removing lysine 56 function by substitution with alanine rendered cells almost insensitive to the residue present at position 57 (Figure 3). Compared to the results obtained with the H3-K56A mutation upon exposure to MMS this result suggests that the role of serine 57 is not as important in the context of transcription elongation as it is in the context of stalled replication fork rescue.

We envisage two types of non-exclusive scenarios to explain our results; modulation of quaternary complexes between histones and their chaperones and modulation of the quaternary structure repertoire of the histone octamer itself. Since our experimental set-up involved constitutive expression of histone H3 mutants, we may have influenced both whilst it may be that the natural modification cycles of lysine 56 and the putative phosphorylation cycle of serine 57 are timed so as to only affect modification of the other residue, histone H3/H4 dimer deposition onto DNA or nucleosome disassembly.

One of the main differences between replication and transcription is probably the absolute requirement to assemble nucleosomes from newly translated histones in the wake of the replication fork, a task that is performed by histone chaperones such as Asf1, CAF-1 and Rtt106 (reviewed in [42], [43]). Notably, these chaperones have also been implicated in transcription elongation [44], [45]. Transcription elongation is known to also rely on nucleosome remodeling involving specific histone modification such as histone lysine acetylation [46] and methylation of H3-K36 [47]. We speculate that, like H3-K56, H3-S57 is involved in some aspect of histone-chaperone interaction [26]. Multiple histone chaperones have been implicated in the H3-K56 acetylation cycle, most notably Asf1p which binds one histone H3/H4 dimer and is absolutely required for H3-K56 acetylation by Rtt109p [48]–[50] which in turn favors histone H3 binding to the Rtt106p and CAF1 histone chaperones [26].

We note that H3-S57 makes a hydrogen bridge with H4-R40 [1], [2] (Figure 5B). Within the nucleosome, helix 1 of histone H4 harbors two pairs of evenly spaced arginines on two of its sides (H4-R35, -R36, -R39 and -R40), of which R39 and R40 are amongst the few individually essential histone H4 residues [51], [39]. Hence, in our experiments, when we substituted serine 57 we may have affected nucleosomal structural dynamics that rely on helix 1 of histone H4, perhaps resulting in altered H4-helix 1 mediated DNA contacts next to the nucleosome's dyad axis of symmetry. Additionally, the putative phosphorylation cycle of histone H3 serine 57 could target RbAP46/48-type histone chaperones during nucleosome remodeling, since these are known to rely on H4-R35, -R36, -R39 and -R40 residues to bind helix 1 of histone H4 [52], [53].
